# Significance of Leaders for Sustained Use of Evidence-Based Practices: A Qualitative Focus-Group Study with Mental Health Practitioners

**DOI:** 10.1007/s10597-019-00430-8

**Published:** 2019-06-12

**Authors:** Karina M. Egeland, Mona-Iren Hauge, Torleif Ruud, Terje Ogden, Kristin Sverdvik Heiervang

**Affiliations:** 1 Division of Mental Health Services, Akershus University Hospital, Sykehusveien 25, Lørenskog, 1478 Norway; 2 Institute of Psychology, University of Oslo, Forskningsveien 3A, Oslo, 0373 Norway; 3 University of Oslo, Institute of Clinical Medicine, Oslo, Norway; 4 Norwegian Center for Child Behavioral Development, Essendropsgate 3, Oslo, 0368 Norway

**Keywords:** Autonomy, Monitoring philosophy, Illness management and recovery, Implementation, Leadership

## Abstract

Evidence-based practices that are implemented in mental health services are often challenging to sustain. In this focus-group study, 26 mental health practitioners with high fidelity scores were interviewed regarding their experiences with implementing the illness management and recovery, an evidence-based practice for people with severe mental disorders, in their services and how this could influence further use. Findings indicate that high fidelity is not equivalent to successful implementation. Rather, to sustain the practice in services, the practitioners emphasized the importance of their leaders being positive and engaged in the intervention, and hold clear goals and visions for the intervention in the clinic. In addition, the practitioners’ understanding of outcome monitoring as a resource for practice improvement must be improved to avoid random patient experiences becoming the decisive factor in determining further use.

**Trial registration**: ClinicalTrials.gov NCT02077829. Registered 25 February 2014.

## Introduction

The implementation of evidence-based practices in mental health services requires much effort. Pursuing high fidelity, that is the degree to which one follows the intervention as specified, has gained considerable attention as it has been associated with positive outcomes, including improved staff retention (Aarons et al. 2009) and patient outcomes (Bartholomew and Kensler 2010; Hasson-Ohayon et al. 2007). However, even for services with high fidelity score, the implementation has shown to be discontinued (Bond et al. 2014; Salyers et al. 2009). The decisions of practitioners to sustain (continue use after the implementation period) or end the use of a practice after its initial adoption are not well understood (Bond et al. 2014; Greenhalgh et al. 2004). Learning from high-scoring fidelity practitioners may provide us with knowledge concerning the challenges faced by the further use of such implementation in their services.

The intervention that form the basis for this study, the illness management and recovery (IMR), is a standardized psychosocial intervention designed to help people with severe mental illnesses manage their illness and achieve personal recovery goals (Mueser et al. 2006). Five strategies form the basis of the IMR intervention: psychoeducation to improve knowledge of mental illness, relapse prevention, behavioral training to improve medication adherence, coping skills training to reduce the severity and distress of persistent symptoms, and social training to strengthen social support. The intervention consists of a combination of educational, motivational, and cognitive-behavioral techniques. By the use of a workbook with educational handouts, practitioners teach patients weekly for 10–12 months, either individually or in groups. A toolkit has been developed together with fidelity checklists for the practitioners to be guided in the implementation of IMR (SAHMSA 2009a, b).

In this paper, implementation is understood as “a specified set of activities designed to put into practice an activity or program of known dimensions” as suggested by (Fixsen et al. 2005, p. 5). A number of conditions have been singled out as influencing whether the implementation of new interventions such as IMR are successful or not. The framework introduced by Fixsen and colleagues (Fixsen et al. 2013; Fixsen et al. 2009) emphasize three drivers, that is *competency drivers*, which involve the selection, training and coaching of practitioners, *organizational drivers* which involve facilitative administrators who prepare and support the use of new interventions, and *leadership drivers*, which involve adaptive leaders who facilitate and support the implementation. In addition, fidelity assessments are seen as a critical component of implementation as they can monitor continuity of practice (Fixsen et al. 2005).

Practitioners play a central role in the implementation of new practices in terms of their attitudes and behavior (Aarons et al. 2011), and also in terms of their autonomy, i.e., whether practitioners perceive independence in how they perform tasks and operate independently. The insertion of new interventions can lead healthcare practitioners to experience the loss of job autonomy and subsequently to increased staff turnover (Aarons et al. 2012; Rossen et al. 2016). However, the implementation of new practices has also shown to lead to increased job autonomy as it gives practitioners more structure and competence in their work (Aarons et al. 2012). High autonomy is therefore emphasized (Greenhalgh et al. 2004; McGuire et al. 2015). Moreover, a difference has been pointed out between practitioners’ *feelings of being autonomous* (i.e., self-regulating, oriented toward the interest value of the environment and contextual supports for self-initiation) and *autonomy support* (i.e., the leaders understanding and acknowledging the perspectives of the practitioners, offering them opportunities to make choices and encouraging self-initiation), both of which have been associated with better job performance (Baard et al. 2004).

The relationship between practitioners and leaders will also affect the implementation of new interventions (Greenhalgh et al. 2004). Similarly to Fixsen et al. (2013), many researchers have argued that leadership is an important determinant for implementation success (Grant et al. 2014; Green et al. 2013; McGuire et al. 2013). Some have highlighted the importance of strong and active leaders who are committed to the entire implementation process for successful intervention (Bond et al. 2014; Rychener et al. 2009; Salyers et al. 2009). A few studies have shown that perceived leader support has been associated with practitioners’ participation in implementation (Sloan and Gruman 1988) and is a significant predictor of a strong climate for implementation (Klein et al. 2001), yet research is needed which explores the specific behavior that leaders may enact in order to facilitate the implementation of interventions (Aarons et al. 2014). A measure was recently developed that differentiated between four types of leader behaviors that are thought to influence implementation; *proactive* leaders who address implementation challenges, *knowledgeable* leaders who understand implementation issues, leaders’ *support* of practitioners’ adoption and use of practices, and *perseverant* leaders who are consistent, unwavering, and responsive to implementation challenges and issues (Aarons et al. 2014).

Practitioners will also be affected by their patients (Greenhalgh et al. 2004). When they experience an innovation as meaningful, which also appears to be meaningful for the patients, this can have a powerful influence on the decision to adopt the practice (Salyers et al. 2009; Stewart and Chambless 2007).

Similarly to Fixsen et al. (2013), many have highlighted the importance of fidelity and outcome monitoring (Miller et al. 2017). Bond et al. (2009) emphasized a “monitoring philosophy” that is making use of regularly monitoring to guide the further implementation. They suggested that introducing fidelity monitoring to services would provide practitioners with a focus for implementation efforts and a framework for understanding the practice. It would also provide leaders with political documentation and offer validation to teams that achieve high fidelity. While few services embraced the fidelity monitoring philosophy, those that did were more likely to sustain the practice. Thus, the practitioners’ understanding must be worked on for them to experience assessments as a resource for long-term sustainability (Bond et al. 2014; Rychener et al. 2009).

In this qualitative focus-group study, a group of practitioners who implemented IMR was interviewed in which the majority had reached high levels of fidelity, which is the exception rather than the rule (Bartholomew and Kensler 2010; Hasson-Ohayon et al. 2007). A previous study with the same practitioners had also shown that the majority wanted to continue to use IMR after the implementation period (Egeland et al. 2017). The aim was to gain more knowledge concerning the practitioners’ experiences with the implementation of IMR in their services, and how this may influence further use. To our knowledge, no one has previously examined such a select group of high IMR fidelity-scoring practitioners. Qualitative research can contribute to deepening and contextualizing our understanding of the conditions of these practitioners, as well as their decisions on whether or not to continue to use IMR.

## Methods

This study was part of a project that implemented IMR in nine Norwegian mental health services (A–I) between November 2013 and June 2015. In addition to a large data collection, the qualitative study consists of nine semi-structured focus group interviews with practitioners who had used IMR for 12 months. The study was approved by the regional committees for medical research ethics [REK 2013/2035].

### Participants

An e-mail was sent to seven primary and six specialized mental healthcare services located in one of Norway’s most populated areas. Seven primary care and two specialized care services agreed to participate in the implementation project. The service leaders were asked to recruit three to five voluntary practitioners at each site (Table 1). Of the 138 employees in the nine participating service settings (*Mdn* = 12 per service, range  9–31), 31 practitioners (22.5%) participated voluntarily in the implementation of IMR. Of these, five were unable to attend the interviews (due to illness or other work responsibilities), leaving 26 (18.8%) practitioners in the nine focus group interviews. The practitioners were mostly female (*n *= 18) and the mean age was 45 years (*SD *= 9.0). The mean years of clinical experience were 12.3 (*SD* = 8.4). The practitioner disciplines included nursing/social education (*n* = 14), social work (*n* = 5), physiotherapy/occupational therapy/pedagogy (*n* = 6), and psychology/medical doctor (*n* = 1). Most of them had a bachelor’s degree (*n* = 22), while some had a master’s degree (*n* = 4). The sample has several similarities to the population of Norwegian public health practitioners, which consists mainly of women (70%) (Statistics Norway 2017b) with a bachelor’s degree (Statistics Norway 2017a). The typical primary care education, in descending order, is in nursing, social education, physiotherapy, and occupational therapy (Norwegian Directorate of Health 2017).

**Table 1:** Service settings and fidelity scores

Service	Number of practitioners	Practitioners^a^	Service setting	Fidelity scores^b^
A	3	Asta^1^ (25), Arve^2^ (6), Ann^1^ (5)	Primary	4.69
B	2	Bob^1^ (14), Brit^1^ (7)	Primary	4.77
C	5	Chris^2^ (6), Christine^1^ (10), Carl^1^ (4), Cate^3^ (4), Celine^2^ (16)	Primary	4.69
D	3	Dagny^1^ (25), Dina^1^ (39), David^2^ (14)	Primary	4.77
E	2	Eline^1^ (10), Erik^1^ (11)	Specialized	–
F	3	Freya^3^ (8), Faye^2^ (12), Frida^3^ (4)	Primary	4.62
G	2	Grete^1^ (13), Gina^4^ (9)	Specialized	4.62
H	3	Hanna^1^ (24), Henrik^1^ (7), Hilda^1^ (9)	Primary	4.46
I	3	Iben^3^ (20), Iris^2^ (5), Iver^3^ (15)	Primary	4.23

Numbers in bracket indicates years of work experience

1 = nurse/social education, 2 = physiotherapy/occupational therapy/pedagogy, 3 = social work, 4 = medical doctor/psychology

^a^The first letter in the practitioners’ alias indicates to which service they belonged

^b^IMR fidelity scale 1–5. The higher the score, the better the fidelity

### Procedure and Materials

An external team of researchers (KE and KH) was responsible for the implementation process and served as an advisory group for the service settings. This engagement throughout the implementation process gave the researchers a wider knowledge and experience with the service settings before the interviews were carried out. A psychologist with extensive experience in IMR was responsible for the training and supervising of the practitioners, which included 4 days of training and weekly telephonic group supervision. An implementation strategy was introduced, while observations and information collection were performed. The strategy included leadership initiation, ongoing training and supervision, fidelity monitoring and feedback after 6 and 12 months, and patient outcome monitoring (the strategy was evaluated in Egeland et al. 2017). The practitioners were asked to get the patients to continuously evaluate their own improvement, which enabled them to pay attention to the patients progression. The practitioners got verbal and written information about the study, and gave consent to participate by signing an informational letter approved by the Norwegian ethical committee (REK).

The interview guide was based on Fixsen et al. (2009) framework on core implementation components (Table 2). This framework was chosen due to its clear overview of central implementation determinants. In addition, two questions were asked about other determinants that hindered or facilitated IMR use. The moderators followed up the practitioners’ responses with further probing questions when appropriate.

**Table 2:** Interview guide

Topics	Sample of questions
IMR experience	What were your experiences with using IMR?
Selection	How was the practitioners recruited to IMR?
Training	Was the training adequate to start practicing IMR? What were your positive and negative experiences with the training?
Coaching	Was the coaching adequate to practicing IMR? What were your positive and negative experiences with the coaching?
Fidelity	How did you experience being interviewed and receiving feedbacks afterwards? Did you work on the feedbacks you received after being evaluated 6 months after startup?
Facilitative administration	What have the administration done to facilitate the IMR practice in the service?
Decision support data system	Have you talked about how to improve and sustain the use of IMR in the future? If so, how would you do this? If not, what are your thoughts about this?
Systems intervention	Have you worked on getting support from the environment around the service? If so, how?
Leadership	How has the leadership been involved in the implementation of IMR?
Hinders/facilitators	Are there other determinants that have hindered/facilitated the implementation of IMR? If so, how?

Focus group interviews were chosen in order to access variations in practitioners’ understanding of the challenges and facilitators of the implementation in the services in which they work. Nine focus group interviews were conducted by one of the authors (KE) who was joined by a co-author (KH) for five of them. Eight interviews were audio recorded and transcribed verbatim. One was analyzed on the basis of written notes due to recording difficulties. The 1 h long interviews took place at the practitioners’ services and were conducted in connection with fidelity monitoring visits. No participant incentives were given. The quotes presented in the results are from the participants, who are identified by alias names to maintain anonymity.

Fidelity was measured on clinic level using the IMR Fidelity scale (McHugo et al. 2007). It assesses how the practitioners in the clinic have implemented specific strategies within the IMR program, such as the use of motivational and cognitive-behavioral techniques, in addition to structural and curriculum-based elements such as the number of sessions held or content modules covered. For the purpose of describing the sample, the services’ fidelity to IMR is included in Table 1.

The services were assessed by the external implementation team during a daylong site visit in which they interviewed leaders, practitioners, and patients after 12 months of implementation. IMR sessions were observed, chart reviews examined, and IMR educational handouts reviewed. The raters independently assessed the program and compared the ratings. Discrepancies were resolved through discussions with each other and with the staff. The service leaders and practitioners received verbal and written feedback, with recommendations for improving implementation. Of the nine service settings, Service E had difficulty implementing IMR. Its practitioners could not recruit patients, and they dropped out of supervision after 7 months. The eight remaining services reached a high score on the IMR Fidelity Scale after 12 months of implementation (Table 1).

### Analysis

All the interviews were audiotaped and transcribed by a professional. As a method for analysis, we have drawn upon the thematic analysis described by Braun and Clarke (2006) and Malterud (2001). This means that when reading through the focus-group interview transcripts, we aimed at an openness and inductive entrance to the data. With regard to interpretation, we leaned towards a realist reading of the data, acknowledging the practitioners’ descriptions, and elaborating and amplifying the meanings that were expressed by the participants. We were concerned with getting closer to the quality and meaning of the practitioners’ experiences as they presented them, seeking to identify patterns and coherences among the participants in the focus group.

The research question that guided the analysis was: “What challenged the practitioners’ further use of IMR?” In the initial reading of the data, the first author and a senior researcher experienced in analyzing qualitative data (MIH) read the transcripts separately. The first author did a close coding of the data, followed by more interpretatively coding of themes that emerged as central in the focus-group interviews, both within and across the interviews. Preliminary themes that emerged were leadership, patient characteristics, and being in time squeeze. In order to make consensus in the analysis, the senior researcher (MIH) independently reviewed the data and noted themes based on the transcripts, followed by in-depth discussions between the two authors. When elaborating themes in the data, some themes were perceived as more prominent of how the practitioners experienced the implementation of IMR in their services and what challenged their further use of the program. For instance, two of the themes that emerged as central in the interviews was the way the practitioners’ notions of whether they would continue to use the program depended on whether they saw it as beneficial for the patients and whether their leaders supported further use in the clinic.

**Fig. 1:**
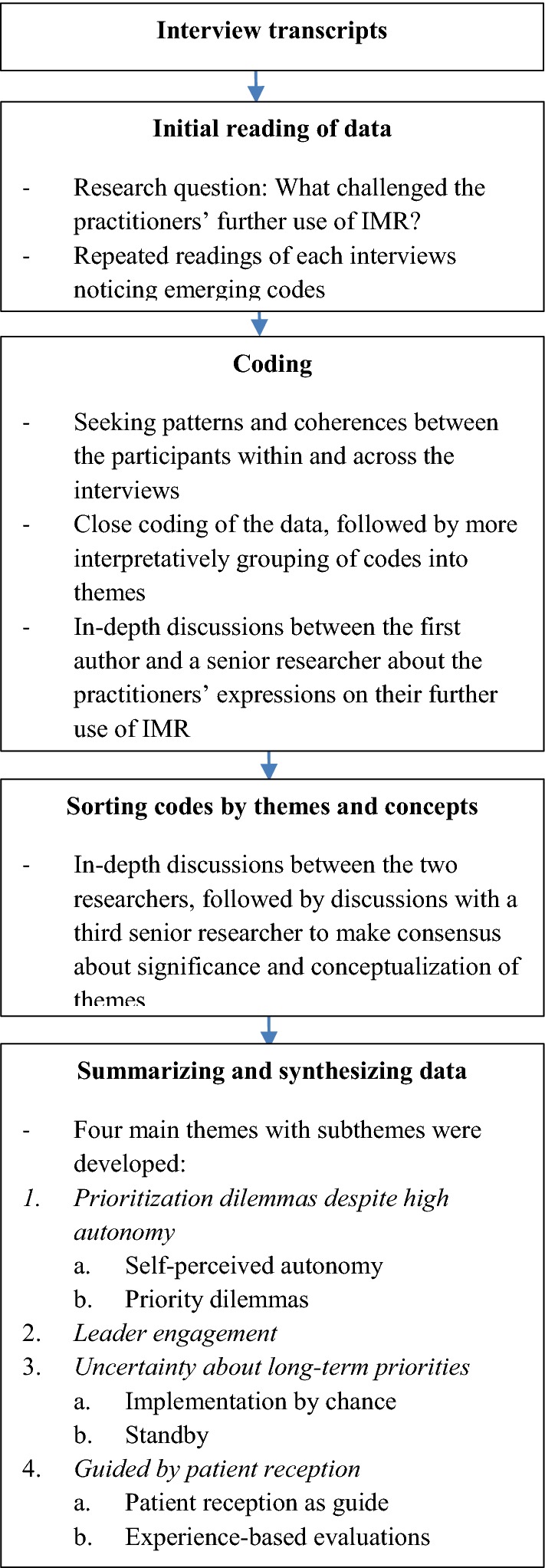
Step-by-step guide for data analysis

In order to select themes of interest and make consensus about significance and conceptualization of themes, the first author had repeatedly discussions with the senior researcher (MIH) throughout the process of analyses. The results of the analysis was also discussed with a third senior researcher with in-depth knowledge of the program and subject field (KH) that participated in five of the interviews. The conceptualization and understanding of the chosen themes was a thorough process, continually questioning of and discussing the connection between the themes and ensuring that they reflected the data. A visual map and subthemes were developed (Fig. 1), and data extracts were selected that illustrated the themes. Simultaneously, themes were connected to relevant theory and literature.

## Results

Most of the practitioners in the study scored high on fidelity during the study, which indicated that they used IMR according to the manual. However, the practitioners expressed views and concerns that might challenge the sustainability of the program in their services, both in terms of maintaining high fidelity levels and in terms of organizational facilitation. Four themes described these challenges: Prioritization dilemmas despite high autonomy, the practitioners’ experiences of leader engagement, uncertainty about long-term priorities, and that practitioners are guided by the patients’ reception of IMR.

### Prioritization Dilemmas Despite High Autonomy

The practitioners in all the participating services expressed a high degree of perceived control regarding how they work. Hanna stated: “I believe that we can choose our methods ourselves. There is no control of details here.” Dagny said: “Our working environment is such that our leader is confident that we are sensible and independent in work, and that we prioritize in a way that benefits the service. We have relatively free reins when we believe in something.” The practitioners chose their own schedule and followed up with their patients according to their clinical competence and their judgement of what was best for their patients. This included their participation in IMR training: All but one of the practitioners, who was requested to do so, chose to attend the training themselves.

Despite feelings of being highly autonomous, the practitioners expressed differences regarding what they believed they could decide for themselves. In terms of reading the IMR manual and preparing for group sessions, some believed that this should be done during their spare time. Faye stated: “We were told to read it, and I did that at home cause I cannot use working hours for that.” Others were determined to not use their spare time. Dagny said: “If I should manage this, I *cannot* use my spare time. I must use working hours.” The practitioners differed in their prioritizing of IMR. Two practitioners expressed that they had no trouble with this. Hanna stated: “I spend 45 min on every session, so I might as well use it on IMR than something else.” However, most of the practitioners seemed to be torn between IMR work and their other responsibilities. Brit stated:

We can control it ourselves, in a way. It’s up to me to let them know that I have too many responsibilities, because I need to work on IMR and that takes time. In the beginning it was like that. I felt the leader was sympathetic. But gradually there has been no need to be considerate toward it. And actually, maybe this has affected the preparation a bit. It has become a bit unstructured.

Although the practitioners showed a faithful use of IMR during the implementation period, they continuously dealt with priority dilemmas throughout their workday. Often IMR was given a lower priority, which might hinder the further use of the model.

In Service C, autonomy was expressed differently. As Carl described it: “This (IMR) has been a priority from Day One, and I have to say the time and free rein we have been given. We don’t feel that we are less prioritized.” The practitioners perceived that they made independent decisions regarding their IMR use, but they seemed to have clarified the work allocations with their staff. They experienced few priority dilemmas between their IMR work and their other responsibilities.

### Leader Engagement

Most practitioners expressed that they felt supported by management in their implementation of IMR. They perceived the leaders as having positive attitudes and showing goodwill toward the program. Brit stated: “She [department leader] has been positive to us, spending enough time to prepare [for IMR sessions]. And the organizational manager, to the extent he understood the content of it, he was also positive toward it.” Service H lacked a leader for most of the period, so Hanna worked as an IMR champion and motivator for her colleagues. When explaining the content of IMR to the new leader, she perceived that he was positive toward it. Thus, positive leaders were receptive of the practitioners’ need to discuss IMR, but did necessarily not bring IMR up themselves. However, practitioners in two other services expressed a lack of leader support. In Service E, although the leader did not demonstrate negativity toward the program, Erik felt that: “they were not backed up properly”. Service G was the opposite; the leader was negative, but trusted the practitioners’ choices. Gina said: “My leader hasn’t been very positive. I had to justify it a little, but at the same time she showed me confidence: If you believe in this, then do it”. Thus, leaders that showed lack of support were unavailable for discussing the content of IMR or the challenges of implementing it.

Despite support from the leaders, there were few indications of them being especially engaged in the implementation process, such as giving proactive attention to reducing barriers, taking responsibility, and following up with the practitioners. As Freya explained: “They [leaders] have arranged for us to receive it [IMR training], and given us the responsibility and a free rein. Yeah, there is really nothing more to say about it.” In most services, the practitioners took the primarily responsibility for facilitating IMR use and solving implementation barriers, without questioning the leaders’ role in this. In some services, the practitioners were clearly dissatisfied with the leaders’ lack of involvement. A few expressed that they were frustrated. Dagny stated: “If this should last, I believe that [strategy plan] is what is required. And then someone must want it. It is very tiring when you all the time [have] to work bottom-up. It has to be top-down.”

In Service C, however, the practitioners gave a clear vision of an engaged, dedicated leader who listened to the practitioners’ wishes, followed up with them regularly, and facilitated implementation in the service. The leader was hands-on in the selection of practitioners to attend the IMR training and considered the practitioners’ capacity beyond the use of IMR. The practitioners expressed gratitude for this engagement. Accordingly, there were differences between leader support and engagement in the services.

### Uncertainty About Long-Term Priorities

The practitioners in the services differed regarding their goals and visions for implementing IMR in their organizations. From the beginning, Service C was goal-oriented toward sustaining the program in the organization. The selection of practitioners to participate in the training was a joint decision between the leaders and the practitioners, and was based on their willingness and the service’s structure. The staff had regular meetings to discuss barriers, their progress, and how to obtain support from several municipality levels. IMR was continually used and talked about in the service. The other services were less prepared for the implementation of IMR. The majority of practitioners were randomly selected; they were often asked by a leader or colleague in passing, which sometimes resulted in sending practitioners at the expense of future prospects. David said:

For me it was a question whether it was a point, since I’m soon retiring. I considered it back and forth. But then I thought it’s alright to try something new at the end of my career, and I decided to go for it. I hope I can give something back of what I’ve learned before I leave this place.

Many of the practitioners experienced a minimum amount of initial briefing on the content of IMR, and some were overwhelmed by the workload it caused. The practitioners implementing it in Service B had not considered whether they had patients who met the inclusion criteria or whether they had the capacity to follow patients on a weekly basis for a year. In Service F, IMR was to be offered to all the patients, but there was no implementation plan for how to facilitate this. Many services thematized IMR at personnel meetings without strategic and regularly examination of the challenges involved in its implementation. Accordingly, choices were made without considering how it would affect the sustainability of the services. Overall, most services seemed to have moved hastily into implementation, without having a long-term plan.

The services’ lack of goals and visions for IMR during the implementation process gave the practitioners mixed signals on what effort they were to put into the program. The practitioners of Services A and F had been told that the program was to be expanded, but they did not know the details of this expansion. The practitioners in Service G were waiting for signals regarding whether or not they were allowed to continue using it. In the remaining services, the practitioners were responsible for deciding whether they would continue to use it. Practitioners in two of the services were pessimistic about the further implementation of IMR in their services. Dagny explained:

I have told the leader that we have been offered something that is really unique [IMR training]. And this should be sustained so that we can offer it to the patients in our service. But I think that takes an effort, and that’s where I believe it takes more than two stakeholders to stay forward.

Others were more eager or hopeful. Frida said: “I feel I have come to a point where I’m curious about what will happen next, and how training should be arranged, and where.” The mixed signals concerning the amount of effort they were expected to dedicate to IMR caused many of the practitioners to be idle and to wait for further instructions. Since the practitioners had to dedicate their own efforts to sustaining the use of the practice, this seemed to increase a risk of their IMR use to drift.

### Guided by Patient Reception

The majority of practitioners had positive experiences with IMR after using it for 12 months. The practitioners emphasized the program’s clear structure, which allowed them to discuss difficult, but important topics. The practitioners in Service A discussed this as follows (sequence):

Asta: “They [patients] get ownership.”

Ann: “It is not said, but they are the tools themselves. And at least I see that, by getting this chance [to receive IMR], it is like an abscess that they haven’t, whether that’s because they haven’t dared or had the time, or been able to see that even though they have an illness, they can still live a good life. So there is a lot of hope in this IMR. And I think that, it gives me much hope too, as a practitioner.”

Asta: “It is inspiring to work with.”

The practitioners perceived that IMR helped to promote shared decision-making with the patients. With regard to treatment, it enhanced the patients’ self-determination to set their own goals and work toward them. However, at the same time, most of the practitioners described the program as comprehensive and time-consuming. Some thought this was beneficial, since it covered many topics that were central to the patients’ everyday lives. This allowed the patients to practice what they had learned, and to achieve long-term goals. However, a few practitioners experienced the program as time-consuming to the extent that it demotivated the further use of it.

The practitioners’ experiences with the program were described in relation to how they perceived their patients as benefitting from the treatment. Grete explained:

You get inspired when you are working with a group and experience that it works. And you see progress in the group participants. When you get positive energy afterwards, it is inspiring to make use of it and form a new group. I have already thought of who I would like to recruit the next time.

The practitioners who expressed positive patient experiences described active patient engagement and a clear progression toward recovery. However, one third of the practitioners expressed some difficulties with using IMR in relation to the patients’ reception of it. In Service C, two of the practitioners discussed the challenges that they experienced (sequence):

Carl: “I believe most of them have taken steps. But the lasting change, to experience the benefit of taking steps toward something. But maybe you are ill the next time, and then it is all wasted in a way.”

Cate: “But I think that every time I bring up recovery goals and what steps you should take for next time, it is like they don’t understand what I’m talking about.”

Carl: “No, they are so low-functioning.”

The practitioners did not see any patient changes, and suggested that the program may be too demanding for low-functioning patients. Accordingly, the practitioners’ initial experience with IMR guided their further use of the program. Those with positive patient experiences expressed that they wanted to continue to use the program in their services. However, the practitioners who experienced small or no visible changes in their patients were more reserved toward the program. Some talked about only making use of parts of the program, particularly the first module, which seemed appropriate for most patients. Bob stated: “I’ve been thinking about using parts of IMR. Different modules for different problems. […] Then it doesn’t seem too discouraging for those who maybe perceive it like that in the beginning. To use it on behalf of the patients.” Other practitioners wanted to change the program, for example, by removing the home assignments. In addition, a few had stopped using it as they perceived that their patients did not benefit from it.

The evaluations of whether the patients benefited from the program were based on either the patients’ feedbacks or the practitioners’ own subjective and professional observations, rather than on systematic assessments. Service F described their observational experiences as decisive in terms of what kinds of patients they would recruit. Frida stated: “when we get experience, we know better who will utilize the program”. The practitioners in a couple of services expressed that they were tired of systematic evaluations. Arve said:

I’m so tired of evaluations. Because, I think that we are continuously considering which initiatives are useful for this group we are working with. […] But to repeatedly assessing what the patients think and stuff like that. Yeah, there is so much that claims our time, and we don’t get any resources for it.

None of the practitioners considered systematic monitoring as tools for evaluating IMR.

## Discussion

In this current study we examined a group of health practitioners who were implementing IMR, in which all but one of the services had reached high levels of fidelity and almost all practitioners wanted to continue to use the program (Egeland et al. 2017). This was in contrast to earlier studies with mixed fidelity results (Bartholomew and Kensler 2010; Hasson-Ohayon et al. 2007). The findings showed that the practitioners expressed their positive experiences with IMR, and wanted to continue to use it. Nevertheless, they perceived challenges in sustaining the use of IMR in their services. Despite feelings of being highly autonomous, they experienced a high-demanding job schedule. The practitioners perceived that their leaders were supportive, but there were few indications of leaders being particularly engaged in the implementation process. Most of the services lacked goals and visions for implementing IMR, and the practitioners’ further use of IMR was steered by the patients’ reception of it. This indicates that high fidelity is not equivalent to successful implementation, and that other circumstances in the services threaten the sustainability of IMR.

Autonomy has been emphasized when new practice are to be implemented (Greenhalgh et al. 2004; McGuire et al. 2015), as it is presumed to increase practitioner job motivation and prevent job turnover (Aarons et al. 2012). However, despite feelings of high autonomy, many practitioners in the study reported dealing with priority dilemmas towards using IMR. The findings can therefore be understood as not supportive of earlier studies promoting practitioner autonomy as ways of sustaining innovation use. Instead, they may provide evidence that the practitioners need support and guidance, rather than autonomy, for sustaining the use of IMR.

Furthermore, a distinction has been identified between practitioners’ feelings of being autonomous and leaders’ support of practitioner autonomy, with both being associated with better job performance (Baard et al. 2004). However, instead of their perceived autonomy helping them to increase the use of IMR, the lack of leader support for their autonomy caused them to hesitate and therefore acted as a barrier to the long-term sustainable use of IMR. Therefore, the findings indicate that autonomy support from their leaders must be present for the practitioners’ autonomy to sustain the use of IMR.

In spite of similar fidelity scorings, the practitioners in Service C stood out as a good example. This indicates that themes other than fidelity are of importance in sustaining IMR. In addition, they perceived their leader to be engaged, dedicated, and committed to the entire implementation process, which has been seen as an important determinant (Aarons et al. 2012; Bond et al. 2014; Salyers et al. 2009). This also corresponds to good implementation leadership, as described by Aarons et al. (2014).

Even though most of the practitioners in the other services experienced that they were supported by their leaders, the leaders’ active engagement differed greatly. It seemed as if the leaders who did not actively engage in the implementation process left the responsibility for the follow-up to the practitioners. As a result, there was a difference between passively supportive leaders and actively engaged leaders. While the former made it possible for the leaders to disengage themselves from the process, the latter tied the leaders to the process, compelling them to act. Based on the findings of this study, we hypothesize that leadership requires more than merely supportive leaders in order to influence practice’s sustainability.

Findings showing that practitioners were being influenced by their patient’s reception of IMR supports earlier research showing that patients have an important impact on the practitioners’ further use of practices (Greenhalgh et al. 2004; Salyers et al. 2009; Stewart and Chambless 2007). Although the external team gave systematic evaluations of the patients to the practitioners, many practitioners based their decisions regarding IMR on a limited sample and displayed a great deal of confidence in their own judgment. Adaptations to interventions are common and sometimes necessary to sustain them. To figure out which pieces of the practice to keep, which patients it would work for, how to recruit and engage them, and how to fit it into their usual workload. This is not necessarily just signs of poor likelihood of sustainability. However, to obtain wanted effect from the intervention, a planned and considered adaptation is necessary, rather than ad hoc (Aarons et al. 2012). Outcome monitoring was not considered as a tool for the further evaluation of IMR, and therefore these practitioners were at risk of rejecting a practice despite good evidence.

As earlier findings (Bond et al. 2009) indicate, the monitoring philosophy, which is considered an important standard in implementation science, seems to be difficult to implement in everyday practice. Similarly to what Rychener et al. (2009) experienced, the practitioners’ understanding must be worked on for them to experience assessments as a resource for program improvement.

The current study used the framework introduced by Fixsen et al. (2013, 2005). Several of the findings support this framework, such as the importance of leadership and fidelity monitoring. Yet, little is said about practitioner autonomy or whether it is guided by patient reception. Therefore, the framework could benefit by emphasizing the health-practitioners’ role in the implementation process beyond the competency drivers that focus on selection, training, and coaching.

There were some strengths and limitations of the current study. The sample consisted of voluntary practitioners in mental health services who achieved high fidelity scores, which is the exception rather than the rule. The results may not be transferable to the implementation of other practices that are required by service leaders or management. The implementation advisory group carried out the interviews, which may have restricted the respondents’ feedback. However, we believe that we managed to reveal both challenges and facilitators in the implementation of IMR in all of the services. In addition, the study was based on the practitioners’ perceptions of the implementation. We do not know to what degree the leaders were engaged or to what degree the practitioners were autonomous. The project was characterized by bottom-up implementation, with voluntary recruitment. The practitioners managed most of the implementation activities, which may have challenged sustainability.

### Implications

The experiences of high-scoring fidelity practitioners who are implementing a new practice have pointed to the importance of the leader’s role in the implementation process. The findings imply that practitioners need leaders who are supportive, actively engaged, and who promote the practitioners’ autonomy in preparing for the sustained use of IMR in the services. Leaders should make sure that services are well prepared, and have the necessary goals and visions for the implementation of IMR. Moreover, the leaders should explicitly address what is expected of the practitioners, and thereby prevent the practitioners from facing priority dilemmas. In addition, the leaders are advised to promote a “monitoring philosophy” by providing systematic evaluations of IMR. This will increase the probability that IMR will be used with high fidelity, in addition to ensuring the improvement of patient outcomes.
